# A case of mistaken identity: a systematic review, meta-analysis, and reinvestigation of hemotropic *Mycoplasma* spp. infection in *Ctenocephalides felis* (cat flea)

**DOI:** 10.1186/s13071-024-06292-8

**Published:** 2024-05-09

**Authors:** Charlotte O. Moore, Erin Lashnits, Michael Lappin, Jennifer Hawley, Edward B. Breitschwerdt

**Affiliations:** 1 Department of Clinical Sciences, North Carolina State University, Raleigh, NC USA; 2 Department of Comparative Biomedical Sciences, School of Veterinary Medicine, University of Wisconsin-Madison, Madison, WI USA; 3 Department of Clinical Sciences, Colorado State University, Fort Collins, CO USA

**Keywords:** *Ctenocephalides felis*, Cat flea, Hemotropic mycoplasma, Vector-borne, Flea-borne pathogen, *Mycoplasma haemofelis*, *Candidatus* Mycoplasma turicensis, *Mycoplasma haemominutum*

## Abstract

**Background:**

Feline-associated hemotropic *Mycoplasma* (hemoplasmas) are believed to be transmitted by two primary mechanisms: (1) direct transmission via fighting and (2) vector-borne transmission by the cat flea (*Ctenocephalides felis*). While the efficiency of transmission by *C. felis* appears low, most manuscripts focus on the prevalence of hemoplasmas in wild-caught fleas and report either a very low (< 3%) or a high (> 26%) prevalence. Therefore, we aimed to assess the influence of sample processing and PCR methods on *C. felis* hemoplasma infection prevalence.

**Methods:**

A systemic review of PubMed articles identified 13 manuscripts (1,531 fleas/flea pools) that met the inclusion criteria (performed PCR for >1 hemoplasma on *C. felis* collected from cats). Risk of bias was assessed utilizing the ROBINS-E tool. Meta-analysis performed in R of these manuscripts found that not washing samples and a common set of 16S rRNA primers first published in Jensen et al. 2001 were associated with increased hemoplasma prevalence. To evaluate the influence of washing on newly collected fleas, we assessed the hemoplasma status of 20 pools of 5 *C. felis* each, half of which were washed and half not washed.

**Results:**

Flea washing did not influence the detection of hemoplasma but instead amplified *Spiroplasma*. To assess non-specific amplification with the Jensen et al. 2001 primers, 67 C. *felis* samples (34% previously reported hemoplasma infected) were subject to PCR and sequencing. By this method, hemoplasma was detected in only 3% of samples. In the remaining “hemoplasma infected” fleas, PCR amplified *Spiroplasma* or other bacteria.

**Conclusions:**

Therefore, we concluded that hemoplasma infection in *C. felis* is rare, and future flea prevalence studies should sequence all positive amplicons to validate PCR specificity. Further investigation of alternative methods of feline-associated hemoplasma transmission and the ability of *C. felis* to maintain hemoplasma infection is necessary.

**Graphical Abstract:**

**Figure:**
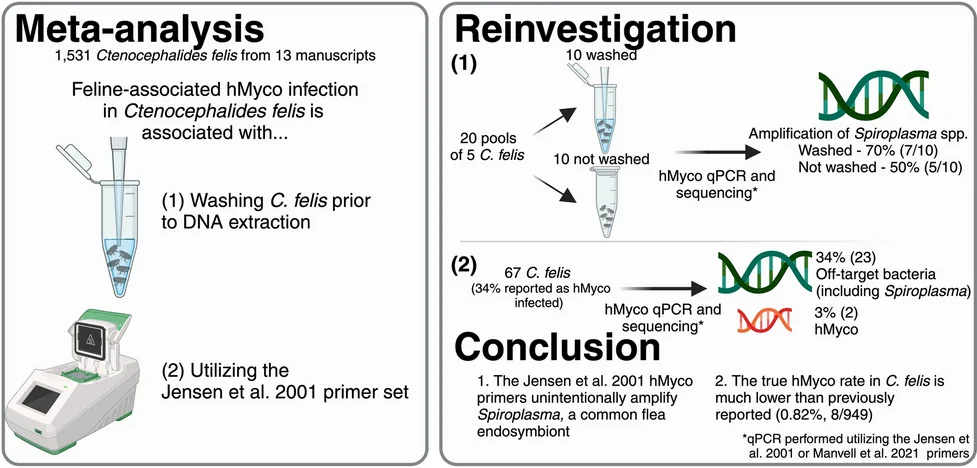

**Supplementary Information:**

The online version contains supplementary material available at https://doi.org/10.1186/s13071-024-06292-8.

## Background

While multiple species of hemotropic *Mycoplasma* (hemoplasma) have been described in domestic and wild animals (e.g. domestic cat, dog, and swine), their presence, disease manifestations, and mechanism of transmission remain incompletely understood [1–5]. Case reports document human infection and disease caused by a human-associated hemoplasma species (*Candidatus* Mycoplasma turicensis) as well as hemoplasma associated with domestic animal species such as the cat (*Mycoplasma haemofelis*), dog (*Candidatus* Mycoplasma haematoparvum), pig (*Mycoplasma suis)*, and sheep (*Mycoplasma ovis)* [4–9]. Human illness varies in reported severity with symptoms that range from pyrexia and hemolytic anemia to lymphadenomegaly and hepatosplenomegaly. Understanding domestic animal-associated hemoplasma transmission and disease manifestations in the classically associated host species will further inform our understanding of disease epidemiology, pathogenesis, and potential zoonotic transmission routes.

The domestic cat is host to three hemoplasma species: *Mycoplasma haemominutum* (Mhm), *M. haemofelis* (Mhf), and *Candidatus* Mycoplasma turicensis (Mtc) [10]. Within the feline host, hemoplasmas infect erythrocytes, occasionally causing hemolytic anemia and fever. Experimental infection documents a spectrum of disease severity, with cats generally manifesting a single episode of transient anemia that resolves spontaneously in approximately 50 days [11, 12]. In immunocompromised cats, hemoplasmas are believed to cause more severe regenerative hemolytic anemia and fever and potentially contribute to myeloproliferative diseases in feline leukemia virus (FeLV)-infected cats [11]. Among the three feline associated hemoplasma species, Mhf is the most pathogenic and Mhm is the most common [13, 14]. Infection in the cat is typically associated with male sex, older age, outdoor access, multi-cat homes, and feline immunodeficiency (FIV) infection [15–18].

The mode(s) of transmission for feline-associated hemoplasmas are poorly understood with researchers and veterinarians proposing two primary mechanisms: transmission via “fighting” (blood contact) and flea-borne transmission [10]. The association of hemoplasma infection with male cats, outdoor access, older age, and FIV infection are all cited as evidence that feline hemoplasmas are transmitted via fighting [15–18]. While experimental transmission via fighting is ethically concerning, current experimental transmission models utilize blood transfusion, mimicking transmission via blood-to-blood contact [13, 19].

In the last 13 years, there has been minimal advancement in the understanding of fleas as vectors for hemoplasma transmission [10]. Based on experimental transmission, *Ctenocephalides felis* (the cat flea) are capable of transmitting Mhf to uninfected cats at a low transmission efficiency (1/6) following infestation with 100 *C. felis* from a hemoplasma-infected cat [14]. However, Mhm was not apparently transmitted by flea infestation in this model, and neither Mhf nor Mhm was transmitted by ingestion of infected fleas [14, 20]. Confusingly, the prevalence of hemoplasma infection in *C. felis* samples varies widely among publications (0–72%), with no evidence of geographic, host, or flea factors to explain this divergence [21, 22]. Assessing the persistence of infection in *C. felis*, dissemination in *C. felis* tissues, and true rate of hemoplasma infection in *C. felis* is necessary to assess the frequency and type (mechanical or biological) of hemoplasma transmission by *C. felis*.

The goal of this study was to reassess the previous reports of high hemoplasma prevalence in wild-caught *C. felis* and determine whether differences in laboratory methods may explain the large range in reported prevalence. To accomplish this, a meta-analysis was performed to assess the influence of specific methodological differences on the prevalence of *C. felis* hemoplasma infection from 13 manuscripts*.* Two variables of interest were included: flea washing and primer set. Flea washing was included because of the observation in reviewing literature that manuscripts including flea washing displayed lower hemoplasma detection. Primer set was analyzed because of the previous non-specific amplification of bacteria other than hemoplasmas with hemoplasma primers (Breitschwerdt Laboratory, unpublished data). This analysis indicated flea washing and the use of hemoplasma primers designed by Jensen et al. 2001 (Myco_Hf_F, Myco_Hf_R) or similar Manvell et al. 2021 primers (Myco_Hf_F.1, Myco_Hf_R) have a significant effect on feline hemoplasma detection in *C. felis* (Table 1) [23, 24]*.* Therefore, the second aim was to experimentally assess whether flea washing removes feline hemoplasma. Following the amplification of bacteria other than hemoplasmas in washed and un-washed fleas utilizing the Myco_Hf_F.1 and Myco_Hf_R primers, the third aim was to reassess the detection of hemoplasma in fleas previously reported as hemoplasma infected with the addition of sequencing to confirm amplicon identities.

**Table 1:** Hemotropic *Mycoplasma* spp. PCR primers. Primers utilized in this study for retesting of hemotropic *Mycoplasma* spp. DNA in stored *Ctenocephalides felis* samples from a previous publication that reported a high hemotropic *Mycoplasma* prevalence in cat fleas [28]

Primer	Oligonucleotide sequence (5′-3′)	Target gene	Reference
Myco_Hf_F	ACGAAAGTCTGATGGAGCAATA	16S hMyco	Jensen et al. 2001 [24]
Myco_Hf_R	ACGCCCAATAAATCCGRATAAT		
Myco_Hf_F.1	GACGAAAGTCTGATGGAGCAAT	16S hMyco	Manvell et al. 2021 [23]
Myco_Hf_R	ACGCCCAATAAATCCGRATAAT		

## Methods

### Meta-analysis of hemotropic *Mycoplasma* spp. in *Ctenocephalides felis*

The evidence for flea-borne transmission of hemoplasmas relies primarily on the detection of hemoplasma DNA in *C. felis* (cat flea) removed from domestic cats. A search on PubMed (https://pubmed.ncbi.nlm.nih.gov/) on December 7, 2023, for five terms (“mycoplasma flea,” “hemobartonella flea,” “hemotropic mycoplasma flea,” “feline hemotropic mycoplasma vector,” “hemoplasma flea”) was utilized to identify suitable articles. The authors also reviewed all citations within the included manuscripts and did not identify additional suitable articles. Articles were included if they tested *C. felis* from cats for more than one hemoplasma species. Manuscripts dated before December 2023 were considered. Abstracts for all manuscripts were reviewed to determine inclusion by the first author (COM) and full texts utilized to collect data. All data were collected by the first author of this manuscript (COM). The total number of fleas, number of hemoplasma-infected fleas, primers utilized for PCR, and use of flea washing was determined for each manuscript. Only manuscripts with all variables of interest reported were considered. If the article included testing both *C. felis* and *Ctenocephalides canis,* only *C. felis* data were selected, if the article gave sufficient information.

Potential sources of bias were considered. Only manuscripts written in English were returned by the search. As the outcomes analyzed were not due to intervention (i.e. assignment to treatment groups), the risk of reporting bias is considered minimal. The greatest potential risk of bias was the inability to locate manuscripts reporting fleas not infected with hemoplasma species due to the use of alternative keywords (e.g. *Bartonella, Rickettsia*) more in line with the study’s findings. Bias due to missing results was unlikely, as all manuscripts were required to report the variables of interest. The ROBINS-E tool (riskofbias.info/welcome/robins-e-tool) was used to guide the assessment of individual study bias (Additional file 1).

Methods of heterogeneity were analyzed as the variables of interest, specifically primer set and flea washing. The outcome of interest was the proportion of hemoplasma-infected fleas. Inclusion criteria required that all studies were eligible for synthesis, as they reported the variables and outcome of interest. Pearson's Chi-squared test was used to assess the association of primer and flea washing with flea hemoplasma infection. In comparisons with > 2 categories, pairwise comparison between proportions was performed to determine significantly different categories. All data analysis and visualization were performed in R (version 4.3.0). A sensitivity analysis was performed including the one manuscript that met almost all inclusion criteria but only tested *C. felis* for Mhf. Certainty in the body of evidence was assessed by study design.

### Effect of washing on hemoplasma status

To assess the effect of flea washing on hemoplasma detection in fleas, 100 previously collected *C. felis* were obtained from free-roaming cats in the Northeast and Midwest US by the FleaNet Biobank (University of Wisconsin-Madison School of Veterinary Medicine, https://fleanet.vetmed.wisc.edu/). Fleas were then assigned to 20 different flea pools (5 fleas per pool). Each flea from an individual cat was assigned to a different pool. The 20 flea pools were then randomized to two treatment groups: washing vs. not washing. For the washing group, half of the flea pools (*n* = 10) underwent two phosphate-buffered saline (PBS) and two 100% ethanol washes, similar to the manuscripts included in the meta-analysis that utilized a wash step [24–26]. Briefly, 200 ul PBS or ethanol was pipetted into the pool of fleas, gently vortexed, and pipetted off. All flea pools then underwent DNA extraction and PCR targeting the 16S rRNA hemoplasma gene utilizing the Myco_Hf_F.1 and Myco_Hf_R primers [23]. These primers were selected because of their prior use and validation within the laboratory. All PCR-positive samples were sequenced and compared to published sequences utilizing NCBI BLAST.

### Reinvestigation of hemoplasma detection in *Ctenocephalides felis*

To reevaluate the prevalence and sequence identity obtained from fleas previously reported as hemoplasma positive, the DNA from 67 individual fleas from a previous survey of hemoplasma in Thailand was provided by a co-author (M.L.) [28]. Conventional PCR utilizing the Myco_Hf_F and Myco_Hf_R primers was first performed at Colorado State University (CSU), and then qPCR utilizing the Myco_Hf_F.1 and Myco_Hf_R primers was performed at North Carolina State University (NCSU). Resulting amplicons at both universities were submitted for sequencing and compared to published sequences utilizing NCBI BLAST. DNA was maintained at −80 °C at CSU and shipped overnight on ice to NCSU where it was stored in a −80 °C freezer.

## Results

### Meta-analysis of hemotropic *Mycoplasma* spp. in *Ctenocephalides felis*

**Fig. 1:**
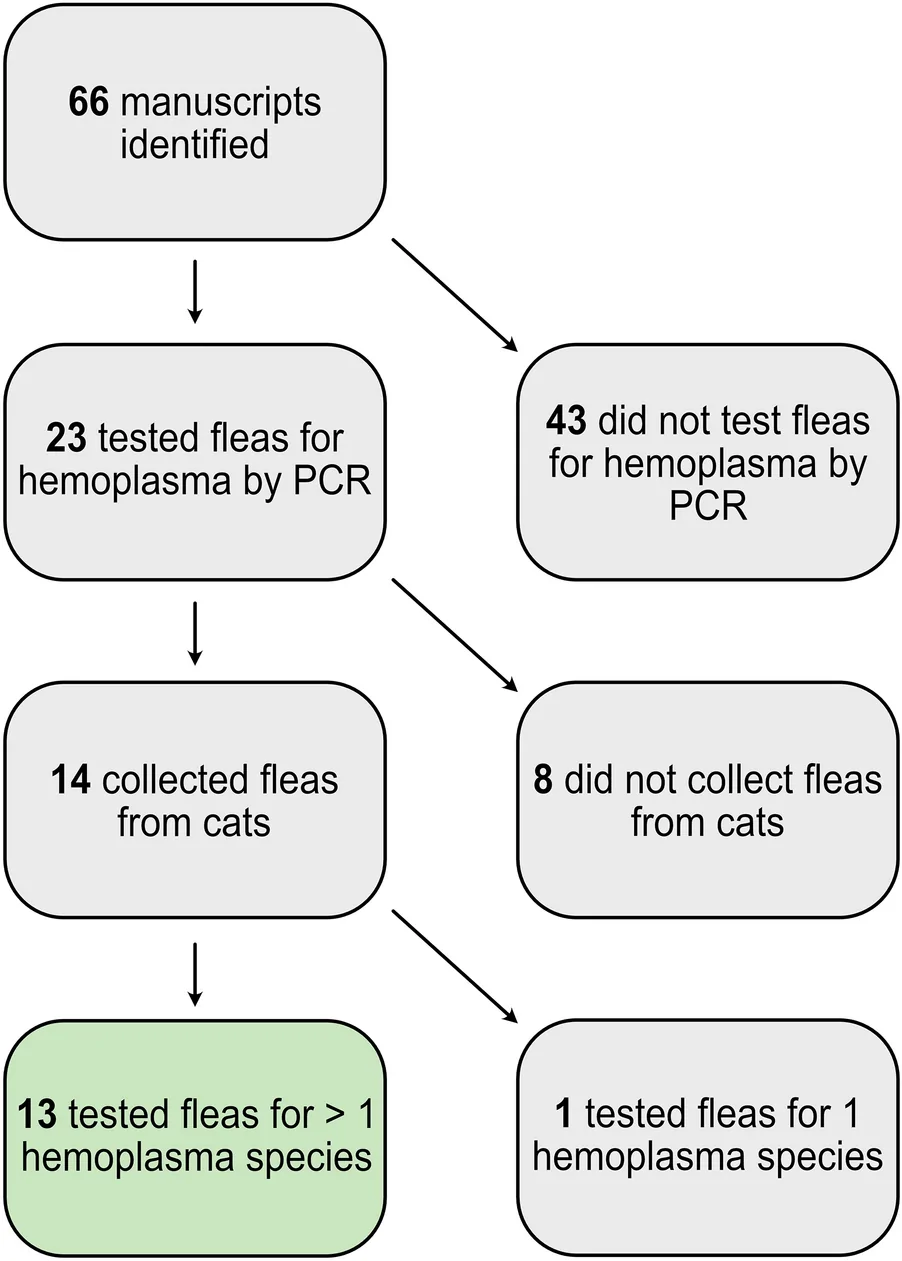
Study selection. Flowchart depicting the number of records identified by our search, reason for exclusion, and number of included records (box shown in green).

The search yielded 66 articles, 13 of which met the inclusion criteria (Fig. 1; Table 2). No abstracts or non-English articles were returned from the search, and all manuscripts that did not include testing of *C. felis* from cats for two or more hemoplasma species were excluded. One manuscript reports the testing of fleas from cats for hemoplasma but only included Mhf and was therefore not considered in the analyses [29]. Citations within the included manuscripts did not identify additional suitable articles.

**Table 2:** Manuscripts reporting hemotropic *Mycoplasma* spp. in wild-caught *Ctenocephalides felis*. Manuscript first author and citation, sample type (pooled or individual fleas), number of fleas per pool, percentage of hemoplasma-positive samples, country of flea origin, and primer set utilized to assess hemotropic *Mycoplasma* spp. presence in wild-caught *C. felis.* All studies that pooled fleas included fleas from a single host animal in the pool. ‘N/A’ indicates that the manuscript did not utilize pooling. ‘Not indicated’ indicates that the manuscript did not report the range of fleas included in flea pools

Manuscript [citation]	Sample type	Fleas per pool	% Hemoplasma-positive samples ± 95% CI (#/Total Tested)	Country	Primer [citation]
Shaw et al. 2004 [30]	Pooled fleas	1–5	49% ± 1.09% (44/90)	UK	Jensen et al. 2001 [24]
Lappin et al. 2006 [31]	Pooled fleas	1–14	27% ± 0.95% (25/92)	US	Jensen et al. 2001 [24]
Willi et al. 2007 [21]	Pooled fleas	Not indicated	3% ± 0.44% (2/73)	Switzerland	Willi et al. 2005 [19]
Kamrani et al. 2008 [18]	Pooled fleas	1–5	72% ± 1.76% (36/50)	Ontario, Canada	Jensen et al. 2001 [24]
Barrs et al. 2010 [22]	Pooled fleas	2–15	56% ± 0.88% (62/111)	Australia	Jensen et al. 2001 [24]
Hornok et al. 2010 [27]	Pooled fleas	1–11	2% ± 0.13% (3/187)	Central-Eastern Europe	Willi et al. 2005 [19]
Assarasakorn et al. 2012 [28]	Pooled fleas	1–3	34% ± 1.86% (17/50)	Thailand	Jensen et al. 2001 [24]
Persichetti et al. 2016 [25]	Pooled fleas	2–5	0% (0/28)	Italy	Martínez-Díaz et al. 2013 [32]
Abdullah et al. 2019 [33]	Pooled fleas	1–89	1% ± 0.03% (3/470)	UK	Peters et al. 2008 [34]
Mifsud et al. 2020 [35]	Pooled fleas	2–5	38% ± 4.53% (8/21)	Thailand	Jensen et al. 2001 [24]
Manvell et al. 2021 [26]	Individual fleas	N/A	0% (0/168)	US, UK	Manvell et al. 2021 [23]
Azrizal-Wahid et al. 2021 [36]	Individual fleas	N/A	0% (0/100)	Malaysia	Criado-Fornelio et al. 2003 [37]
Zarea et al. 2022 [38]	Individual fleas	N/A	0% (0/91)	Asia	Tasker et al. 2010 [39]

Manuscripts were diverse, including studies from Asia, Australia, Europe, and North America and spanning from 2004 to 2022. Most manuscripts utilized primers targeting the hemoplasma 16S rRNA gene developed by Jensen et al. 2001 (Myco_Hf_F and Myco_Hf_R) or similar Manvell et al. 2021 primers (Myco_Hf_F.1 and Myco_Hf_R) [23, 24]. The Myco_Hf_F.1 primer is almost 100% homologous to the Myco_Hf_F primers but has a one base pair shift in the forward primer that is homologous to hemoplasma and *Spiroplasma* spp. sequences (Table 1). In total, these 13 manuscripts reported PCR results from 1531 *Ctenocephalides* fleas or flea pools (Additional file 2).

Risk of bias, as assessed by the ROBINS-E tool, was considered uniformly low for all manuscripts due to the meta-analysis inclusion criteria and observational nature of all studies. Methodological differences are considered the most likely source of misclassification error and are therefore the focus of analysis.

The first methodological variation of interest was the washing procedures implemented among different studies. Manuscripts that implemented washing (*n* = 3) reported 0.78% (3/383) of *C. felis* infected with hemoplasmas whereas manuscripts that did not implement washing (*n* = 10) reported 17.2% (197/1148) (*p* < 0.0001; Pearson’s Chi-squared test). Upon further analysis, the primer set utilized by these manuscripts appeared to be a confounding variable.

Comparing the hemoplasma detection rate of manuscripts utilizing the Myco_Hf_F/Myco_Hf_F.1 and Myco_Hf_R primers to those utilizing other primer sets, seven manuscripts were identified that utilized the Myco_Hf_F/Myco_Hf_F.1 and Myco_Hf_R primers and six manuscripts utilized other primer sets. Manuscripts utilizing the Myco_Hf_F/Myco_Hf_F.1 and Myco_Hf_R primers reported 33% (192/582) of *C. felis* as hemoplasma infected compared to 0.82% (8/949) of *C. felis* infected in manuscripts utilizing other primer sets (*p* < 0.0001; Pearson’s Chi-squared test). Of the manuscripts utilizing the Myco_Hf_F/Myco_Hf_F.1 and Myco_Hf_R primers, three reported sequencing all samples and one reported sequencing Mhf or Mtc positive samples. In those that did not utilize sequencing, hemoplasma identity was assessed by amplicon size via gel electrophoresis. Of the three manuscripts reporting sequencing, only one reported the GenBank accession ID of sequences.

When assessing the two variables of interest (washing and primer set), fleas from manuscripts that utilized the Myco_Hf_F/Myco_Hf_F.1 and Myco_Hf_R primer set and did not wash fleas clearly displayed the highest hemoplasma infection prevalence (Table 3; *p* < 0.0001). Therefore, further investigation of unwashed fleas and confirmation of the hemoplasma detection in fleas tested with the Myco_Hf_F/Myco_Hf_F.1 and Myco_Hf_R primer set were required to determine if increased hemoplasma detection could be attributed to flea washing or primer set. Sensitivity analysis included one additional manuscript that utilized only Mhf PCR and found that all statistical tests yielded the same significant/non-significant result [29]. All studies were descriptive, observational studies. Certainty of prevalence estimates varied across studies based on study size (*n* = 21–111).

**Table 3:** Primer set, flea pooling, and flea washing are associated with detection of hemotropic *Mycoplasma* spp. This table displays the percentage of hemotropic *Mycoplasma*-infected fleas reported by the identified manuscripts based on the PCR primers utilized and whether authors utilized a washing step prior to DNA extraction. Primers are grouped as either those designed by Jensen et al. 2001 or other primers. *Categories that were significantly different from all other categories by pairwise comparison of proportions post hoc test. *p < 0.05, **p < 0.01, ***p < 0.0001

Primer set	Washed	Percentage infected fleas ± 95% CI (# pos/total)
Myco_Hf_F/Myco_Hf_F.1 and Myco_Hf_R	No	46.38 ± 0.24% (192/414) ***
Myco_Hf_F/Myco_Hf_F.1 and Myco_Hf_R	Yes	0% (0/168)
Other primers	No	0.68 ± 0.03% (5/734)
Other primers	Yes	1.40 ± 0.11% (3/215)

### Effect of washing on hemoplasma prevalence

Of the 20 new *C. felis* pools that underwent DNA extraction and PCR for hemoplasma, 12 pools (60%) were positive by PCR including 7 (70%) in the wash group and 5 (50%) in the no-wash group (*p* = 0.65; Fisher’s exact test). However, when sequenced, all amplicons were identified as *Spiroplasma* spp. (GenBank accession no. EU170606.1), not a hemoplasma species.

### Reinvestigation of hemoplasma detection in *Ctenocephalides felis*

DNA from fleas previously reported as hemoplasma positive [28] was obtained and retested utilizing the Myco_Hf_F and Myco_Hf_R primers at Colorado State University and the Myco_Hf_F.1 and Myco_Hf_R primers at North Carolina State University [23, 24]. The original study assessed amplicon identity by gel electrophoresis and comparison to sequence length, not by sequencing. In total, only two *C. felis* (2/67, 3% vs. the previously published 34%) contained hemoplasma DNA, as confirmed by sequencing. *Spiroplasma* spp. DNA was amplified and sequenced from a majority of previously PCR positive samples (Table 4). Non-specific amplification of bacteria other than hemoplasma was confirmed independently at two institutions when using both primer sets (Table 5)*.*

**Table 4:** Results from hemotropic *Mycoplasma* spp. PCR. Percentage and total number of fleas infected with different hemotropic *Mycoplasma* spp. and other bacteria based on hemotropic *Mycoplasma* PCR and sequencing with the Jensen et al. 2001 and Manvell et al. 2021 primers. One sample was positive for *Limosilactobacillus* spp. and *Spiroplasma* spp. and is therefore included in both. If samples were negative by one PCR and positive by another, they were included in only the positive result. The GenBank Accession ID is included for reference

Organism	% DNA positive fleas (#)	GenBank accession ID
*Spiroplasma* spp.	23.9% (16)	EU170606.1
*Mycoplasma haemofelis*	3.0% (2)	AM748929.1
*Limosilactobacillus* spp.	3.0% (2)	OP554566.1
*Sinocapsa zengkensis*	1.5% (1)	NR_177749.1
*Lactobacillus rhamnosus*	1.5% (1)	GU429435.1
*Bacillus cereus*	1.5% (1)	MN251217.1
Uncultured bacterium	4.5% (3)	MF224262.1 (*n* = 2) MT013437.1 (*n* = 1)
Negative	62.7% (42)	N/A

**Table 5:** Comparison of Manvell et al. 2021 and Jensen et al. 2001 hemotropic *Mycoplasma* spp. PCR primers. Percentage of samples (number of samples) with non-specific amplification, hemotropic *Mycoplasma* spp. amplification, or negative PCR results. A small proportion of samples did not have enough excess DNA available for testing utilizing the Manvell et al. 2021 primers and are therefore listed as “Not available for testing.” Primers utilized are those from Manvell et al. 2021 [23] and Jensen et al. 2001 [24]

Manvell et al. 2021	Jensen et al. 2001		
	Non-specific amplification	*Mycoplasma haemofelis*	Negative
Non-specific amplification	22.4% (15)	0% (0)	5.5% (5)
*Mycoplasma haemofelis*	0% (0)	0% (0)	0% (0)
Negative	4.5% (3)	1.5% (1)	59.7% (40)
Not available for testing	0% (0)	1.5% (1)	3% (2)

## Discussion

This manuscript documents the frequent amplification of *Spiroplasma* spp. or other bacteria by the Myco_Hf_F/Myco_Hf_F.1 and Myco_Hf_R primers developed to detect the hemoplasma 16S rRNA gene [23, 24]. This suggests that previous reports of high hemoplasma prevalence in *C. felis* are likely due to misreporting of *Spiroplasma* or other bacteria in the absence of sequencing or primers specific for hemoplasma. Using stored DNA from Assarasakorn et al., hemoplasma was detected in only 3% (2/67) of individual *C. felis* compared to the 34% (17/50) of flea pools reported in the original study [27]. This finding reinforces the importance of sequencing PCR amplicons and comparison to a robust, updated database of sequences.

The finding that flea washing did not influence hemoplasma detection suggests that these methodological changes are not responsible for the variability of hemoplasma detection in the 13 manuscripts analyzed. The failure to detect hemoplasma with or without washing suggests that differing hemoplasma prevalence is not due to physical removal of hemoplasma from the surface or flea mouthparts.

The primary evidence for flea-borne transmission of feline hemoplasmas is the high prevalence in *C. felis*. Therefore, with a lower revised prevalence, we suggest there is limited evidence for *C. felis* facilitating sufficient transmission for the current prevalence of hemoplasma in cats worldwide (~ 20%) [40, 41]. Other epidemiology and experimental study findings provide limited evidence that *C. felis* is primarily responsible for transmission of hemoplasma: (1) poor transmission efficiency in experimental studies, (2) dissimilar hemoplasma and *C. felis* or *Bartonella* spp. (a known flea-borne pathogen) epidemiology, and (3) a lack of correlation between cat hemoplasma infection and flea infestation. Additionally, the findings reported in this manuscript suggest that (4) wild-caught *C. felis* are only occasionally infected with hemoplasmas.

**Fig. 2:**
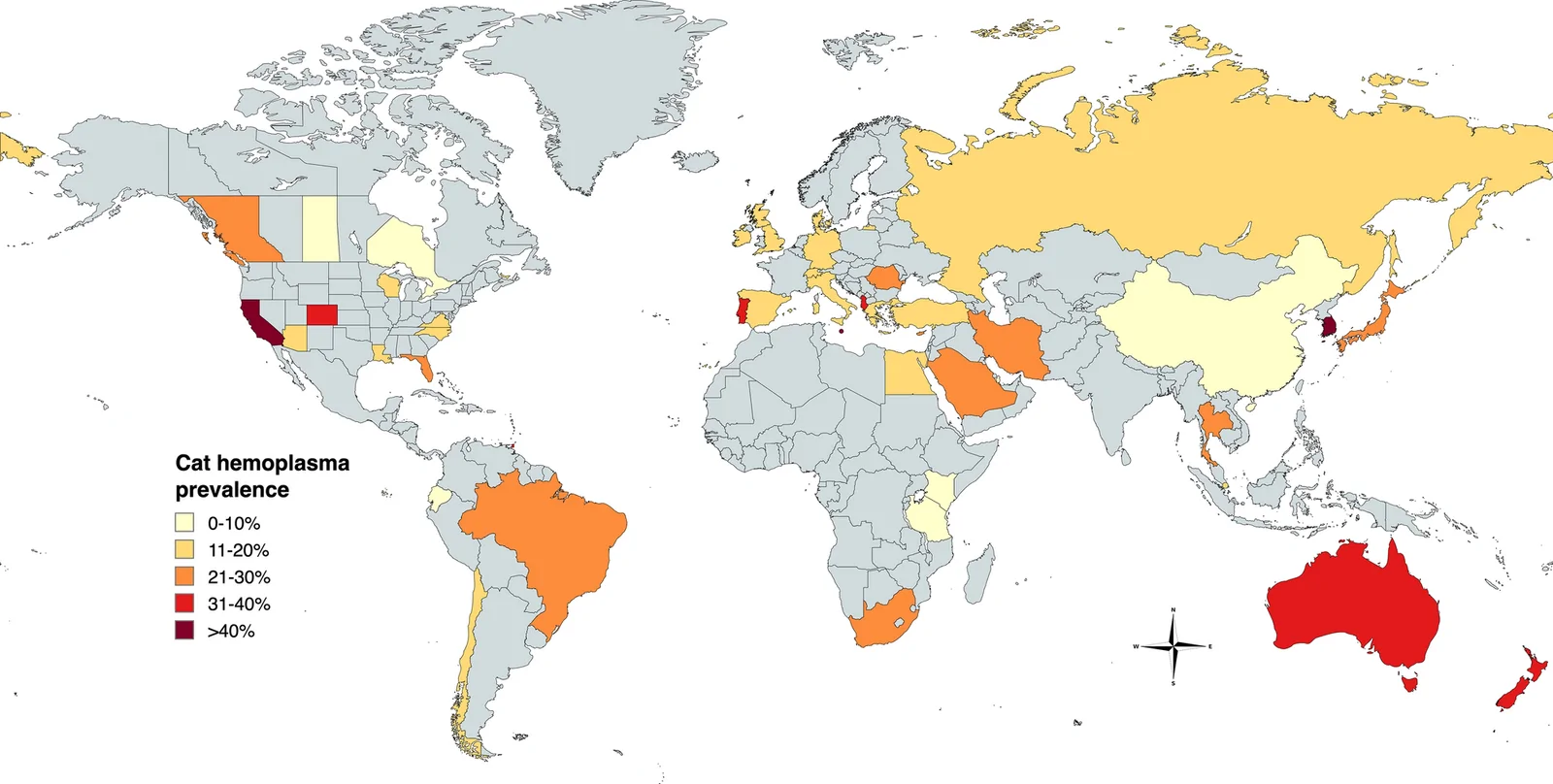
Global epidemiology of feline hemotropic *Mycoplasma* spp. infection. Rate of hemotropic *Mycoplasma* infection from manuscripts obtained from PubMed searching every combination of “[Country] cat mycoplasma.” If multiple manuscripts were obtained from a single country, the prevalence was recalculated based on the number of cats tested and number of hemotropic *Mycoplasma*-positive cats. Map was created utilizing mapchart.net. Prevalence is derived from the following manuscripts: Albania [48], Australia [22, 49], Brazil [40, 50–55], Canada (British Columbia [56], Ontario [18], Saskatchewan [57], Prince Edward Island [58]), Chile [59], China [60], Cyprus [61], Denmark [62], Ecuador [63], Egypt [64], Germany [17, 65], Greece [66, 67], Iran [68], Ireland [69], Italy [25, 45, 70–72], Kenya [29] Japan [46, 73, 74], New Zealand [75], Portugal [32, 76], Qatar [77], Romania [78], Russia [79], Saudi Arabia [80], South Africa [81], South Korea [82], Spain [40, 83, 84], Switzerland [19], Tanzania [28], Thailand [28, 85–87], Turkey [88, 89], Trinidad and Tobago [90], England [91], USA (Arizona [92], California, Colorado, and Florida [93], Louisiana [94], and Virginia and North Carolina [23]), Scotland [95], and Singapore [93]

- (1) While experimental infection of domestic cats by inoculation with hemoplasma-infected blood from a donor cat is well documented, experimental infection by *C. felis* fed on hemoplasma-infected cats failed to transmit hemoplasma to a majority of recipient cats [13, 14, 42, 43]. Hemoplasmas can be detected by PCR in *C. felis* after feeding on an infected cat (Mhm or Mhf); however, the ability of hemoplasmas to colonize *C. felis*, proliferate within *C. felis*, or disseminate to the *C. felis* salivary glands for transmission in the saliva is unknown [14]. In attempted experimental infection by fleas fed on known hemoplasma-infected hosts, the one cat that displayed infection, as indicated by PCR amplification, likely acquired a low hemoplasma dose as they failed to manifest clinical or hematological abnormalities despite infestation with far more infected fleas than is expected to occur in nature [14]. Additional attempts to infect cats by ingestion of infected *C. felis* or *C. felis* feces failed to produce infection [20]. Given the low rate of transmission and a suspected low infectious dose delivered by *C. felis* (too low to develop disease), the authors suggest that *C. felis* may be an occasional mechanical vector of hemoplasmas and an unlikely biological vector.
- (2) The lack of association between feline hemoplasma infection and *C. felis* epidemiology further questions the role of *C. felis* in feline-associated hemoplasma transmission. Compared to predicted *C. felis* prevalence (see Lawrence et al. 2019), feline hemoplasmas do not display similar epidemiology, being overrepresented in certain countries (e.g. Saudi Arabia, Iran, South Korea) and underrepresented in others (e.g. China; Fig. 2) [44]. This lack of association is confirmed by large surveys (*n* > 950 cats) from Italy and Japan that failed to determine geographic variations in hemoplasma presence despite predicted variations in *C. felis* epidemiology [45, 46]. In contrast, in the same study from Italy and a similar study from Japan, a geographic association was detected for feline *Bartonella *spp., a known flea-borne pathogen [45, 47]. This lack of association also extends to the association of host hemoplasma infection with *C. felis* infestation.
- (3) Investigating the association of host hemoplasma infection and flea infestation consistently fails to report an association [32, 39, 40, 77, 80, 85], with limited studies detecting an association [55]. However, due to the persistence of hemoplasma infection over long periods of time and the association of flea infestation with other variables (e.g. outdoor access, owned status), these studies are of limited value to assess the relevance of *C. felis* as a vector of hemoplasma species.
- (4) Finally, our finding that non-hemoplasma bacteria are frequently amplified by primers targeting the 16S rRNA gene, specifically the Myco_Hf_F/Myco_Hf_F.1 and Myco_Hf_R primers, indicates that the true rate of hemoplasma infection in *C. felis* in nature is much lower than previously reported. The frequency of off-target amplification utilizing these primers reflects the importance of sequencing and advantage of utilizing a gene target specific to the genus of interest. Across manuscripts not utilizing the Myco_Hf_F/Myco_Hf_F.1 and Myco_Hf_R primers, the cumulative percentage of hemoplasma-infected *C. felis* is < 1% (0.82%; 8/949). We propose this detection rate may be due to the ingestion of hemoplasmas in the infected bloodmeal and not infection or colonization of the flea. As the hemoplasma infection status of the tested *C. felis* was unknown, it was not possible to assess the effect of washing on hemoplasma infection. However, the authors still encourage washing of vector samples, particularly for microbiome analysis [96].

Limitations of this meta-analysis include a limited body of literature and potential failure to identify manuscripts with hemoplasma-negative fleas due to the use of alternative keywords. Furthermore, this manuscript did not assess the influence of host factors (outdoor access, sex, etc.), rigor of flea collection, or average number of fleas per host on reported hemoplasma prevalence. Experimental work completed in this manuscript is limited by the observational nature with a clear need for experimental transmission studies to fully understand the extent of flea involvement in hemoplasma transmission. Furthermore, testing of known infected *C. felis* with and without flea washing is necessary to assess the influence of washing on pathogen detection.

## Conclusions

In summary, the revised low rate of *C. felis* hemoplasma detection necessitates further investigation of the efficiency of *C. felis* as a vector and ability of *C. felis* to maintain hemoplasma infection. Future investigation of hemoplasma transmission should also explore alternative modes of transmission. Transmission by alternative vector species is considered unlikely given the inability of *Aedes aegypti* to transmit hemoplasma infection and limited detection of hemoplasma infection in other wild-caught ectoparasites (e.g. ticks) [21, 97, 98]. Vertical transmission of hemoplasma is also considered unlikely in the cat because of the infrequent detection in female cats or their reproductive tissue and an association of infection with older age [1, 23]. Based upon current evidence, feline-associated hemoplasmas are likely transmitted by biting and scratching during fighting. This is supported by epidemiological studies that report an association of hemoplasma infection with male cats, older age, and outdoor access [15, 17, 40, 46, 71], as well as experimental studies that report transmission of anemia by ingestion of blood from an anemic cat and the detection of hemoplasmas in cat saliva and salivary glands [99, 100]. Based on this evidence, direct transmission of hemoplasma species via fighting presents a promising area of research.

## Supplementary Information


**Additional file 1: S1.** PRISMA Checklist. Preferred Reporting Items for Systematic Reviews and Meta-Analyses (PRISMA).**Additional file 2: S1.** Data list of articles included in the systematic review and sensitivity analysis.

## Data Availability

The data utilized for meta-analysis are available in the supplementary data file.
