# Correction: A case of mistaken identity: a systematic review, meta-analysis, and reinvestigation of hemotropic Mycoplasma spp. infection in Ctenocephalides felis (cat flea)

**DOI:** 10.1186/s13071-024-06536-7

**Published:** 2024-10-30

**Authors:** Charlotte O. Moore, Erin Lashnits, Michael Lappin, Jennifer Hawley, Edward B. Breitschwerdt

**Affiliations:** 1 Department of Clinical Sciences, North Carolina State University, Raleigh, NC USA; 2 Department of Comparative Biomedical Sciences, School of Veterinary Medicine, University of Wisconsin-Madison, Madison, WI USA; 3 Department of Clinical Sciences, Colorado State University, Fort Collins, CO USA

**Correction: Parasites & Vectors (2024) 17:209** 10.1186/s13071-024-06292-8

Following publication of the original article [1], it was brought to the journal's attention that there was a citation-related error in the bulleted list in the Discussion section of the article. Namely, in points 2–4 of the list, the citations had all been erroneously replaced by citations of reference number 14. In addition, it was flagged to the journal that in place of a color version of Figure 1, a black and white version of the figure had been published. The citations and the figure have since been corrected. The publisher thanks you for reading this erratum and apologizes for this processing error.
